# The effect of fractional inspired oxygen concentration on early warning score performance: A database analysis

**DOI:** 10.1016/j.resuscitation.2019.04.002

**Published:** 2019-06

**Authors:** James Malycha, Nazli Farajidavar, Marco A.F. Pimentel, Oliver Redfern, David A. Clifton, Lionel Tarassenko, Paul Meredith, David Prytherch, Guy Ludbrook, J.Duncan Young, Peter J. Watkinson

**Affiliations:** a Kadoorie Centre for Critical Care Research and Education, Nuffield Department of Clinical Neurosciences, University of Oxford, Level 3, John Radcliffe Hospital, Headley Way, Oxford, OX3 9DU, United Kingdom; b James Black Centre, King’s College London, London SE5 9NU, United Kingdom; c Institute of Biomedical Engineering, Department of Engineering Science University of Oxford, Old Road Campus Roosevelt Drive, Oxford OX3 7DQ, United Kingdom; d Research and Innovation Department, Portsmouth Hospitals NHS Trust, Portsmouth PO6 3LY, United Kingdom; e Centre for Healthcare Modelling & Informatics, School of Computing, University of Portsmouth, Portsmouth PO1 2UP, United Kingdom; f University of Adelaide, Faculty of Health and Medical Science, North Terrace, AHMS Floor 8, 5000, Australia; g NIHR Biomedical Research Centre Oxford, Kadoorie Centre for Critical Care Research and Education, Nuffield Department of Clinical Neurosciences, University of Oxford, Level 3, John Radcliffe Hospital, Headley Way, Oxford OX3 9DU, United Kingdom

**Keywords:** Critical care, Intensive care, Early warning scores, Fractional inspired oxygen, Predictive scores, Machine learning, Decision trees

## Abstract

**Objectives:**

To calculate fractional inspired oxygen concentration (FiO_2_) thresholds in ward patients and add these to the National Early Warning Score (NEWS). To evaluate the performance of NEWS-FiO_2_ against NEWS when predicting in-hospital death and unplanned intensive care unit (ICU) admission.

**Methods:**

A multi-centre, retrospective, observational cohort study was carried out in five hospitals from two UK NHS Trusts. Adult admissions with at least one complete set of vital sign observations recorded electronically were eligible. The primary outcome measure was an ‘adverse event’ which comprised either in-hospital death or unplanned ICU admission. Discrimination was assessed using the Area Under the Receiver Operating Characteristic curve (AUROC).

**Results:**

A cohort of 83,304 patients from a total of 271,363 adult admissions were prescribed oxygen. In this cohort, NEWS-FiO_2_ (AUROC 0.823, 95% CI 0.819–0.824) outperformed NEWS (AUORC 0.811, 95% CI 0.809–0.814) when predicting in-hospital death or unplanned ICU admission within 24 h of a complete set of vital sign observations.

**Conclusions:**

NEWS-FiO_2_ generates a performance gain over NEWS when studied in ward patients requiring oxygen. This warrants further study, particularly in patients with respiratory disorders.

## Key messages

**What is the key question?**

Does adding FiO_2_ to NEWS improve performance when predicting in-hospital death and unplanned ICU admission in ward patients?

**What is the bottom line?**

In hospital ward patients on oxygen therapy, adding FiO_2_ to NEWS results in a significant performance increase (NEWS-FiO_2_: AUROC 0.823, 95% CI 0.819–0.824 versus NEWS: AUROC 0.811, 95% CI 0.809−0.814) when predicting in-hospital death and unplanned ICU admission.

**Why read on?**

This large, multi-centre, cohort study demonstrates how to calculate and then add FiO_2_ thresholds to NEWS and then test performance against NEWS in a separate database.

## Introduction

An Early Warning Score (EWS) identifies clinical deterioration in hospitalised patients using simple algorithms that sum integer scores assigned to values of individual vital sign observations^1^ The score increases as the vital signs become more abnormal. The summed scores are then calibrated against subsequent in-hospital adverse events to generate thresholds to trigger escalations in care.^2^ EWS systems were originally designed to be paper-based, but as some vital sign recording has become electronic, more sophisticated systems have been developed.^3^, ^4^

Most EWS systems use peripheral blood oxygen saturation (SpO_2_) recorded by a pulse oximeter as one of the vital signs. However, patients with a low SpO_2_ are often treated by increasing their inspired oxygen fractional concentration (FiO_2_), which returns their SpO_2_ value to normal and makes the FiO_2_ the important value for detecting deterioration^5^, ^6^ Despite this, FiO_2_ is not part of any widely implemented EWS^7^. Techniques that minimise this information loss by using the fractional inspired oxygen as an alternative or adjunct to SpO_2_ to construct an EWS are relatively under-developed, and are mainly used in obstetric populations^8^, ^9^ The National Early Warning Score (NEWS) is the most widely adopted in the UK and scores in a binary manner for oxygen use (scoring zero for room air and two for all forms of supplementary oxygen).^10^, ^11^ As a result, it may be that important information about respiratory dysfunction in ward patients is being lost in NEWS, impairing its performance. The lack of granularity also means that escalations in oxygen therapy may occur without an increase in score, creating a risk of these changes being missed by reviewers. This study was designed to test the hypothesis that adding FiO_2_ to NEWS would improve the predictive performance of the score when used in patients requiring oxygen.^12^

## Methods

This multi-centre cohort study is reported following the TRIPOD guidelines for the development and validation of predictive models.^13^ The TRIPOD checklist is included in the supplementary digital content **(SDC-1)**. We performed a two-centre, retrospective study using two large databases of routinely collected healthcare data. This study was part of a larger project for which ethics approval had previously been obtained (Health Research Authority reference: Oxford University Hospitals Trust Research Ethics Committee reference 16/SC/0264; confidentiality advisory group: 16/CAG/0066, Isle of Wight, Portsmouth and South East Hampshire Research Ethics Committee reference: 08/02/1394).

### Source of data

One database contained vital signs, oxygen administration data and patient outcome data on all patients admitted to the four acute care hospitals in the Oxford University Hospitals NHS Foundation Trust (OUHNHSFT) between October 2014 and October 2016 where vital sign recordings were taken at the bedside using the System for Electronic Notification and Documentations (SEND, Sensyne Health, www.sensynehealth.com)^14^ The second database contained similar data on patients admitted to the Queen Alexandra Hospital (QAH), an acute care hospital in Portsmouth, between January 2010 and May 2016 where vital sign recordings were taken using CareFlow Vitals (System C Healthcare, www.systemc.com).

### Participants

**Fig. 1:**
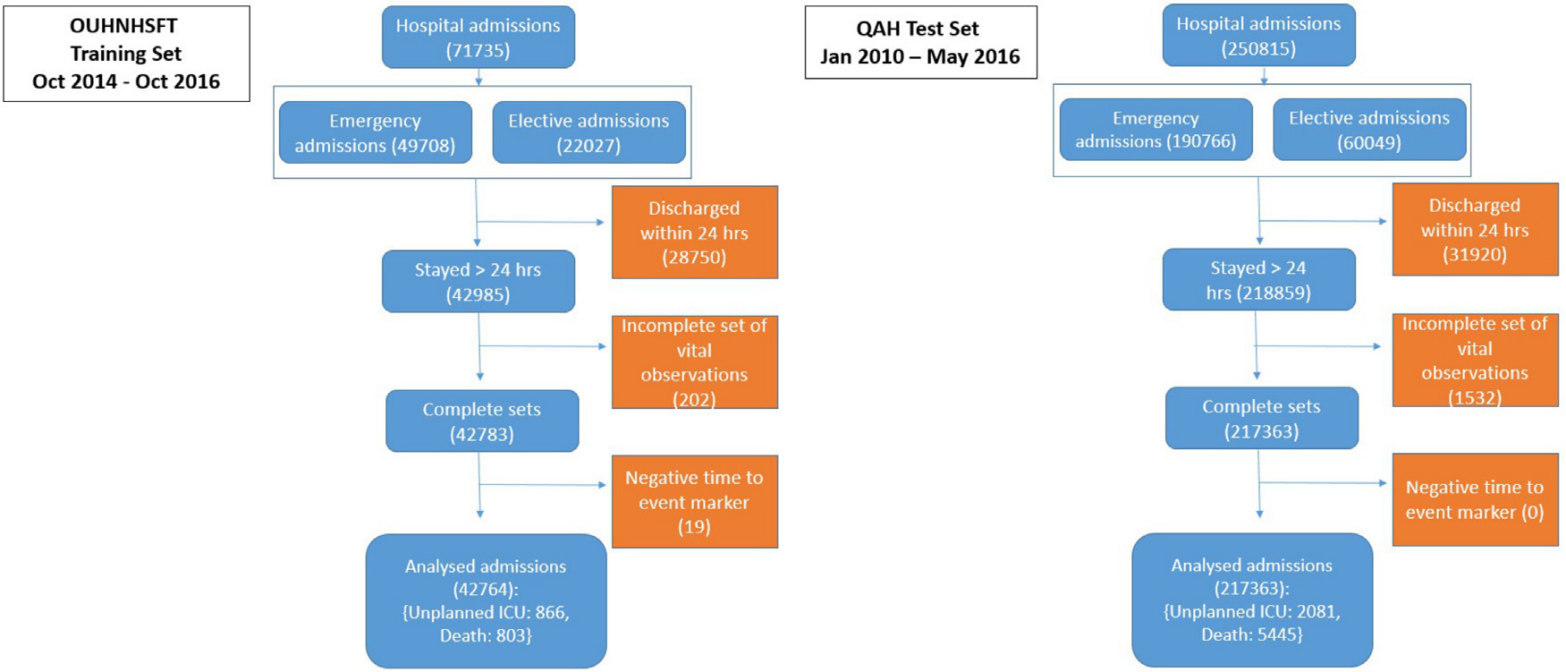
Flowchart of included and excluded patients in both datasets.

All admissions to the OUHNHSFT hospitals and the QAH were eligible for the study. To be included in the analysis, patients were required to be adult (≥16 years of age) at hospital admission, with a hospital stay of ≥24 h with at least one complete vital sign observation set. Vital sign observations sets were only eligible for analysis once the patient reached the ward and having not arrived there via ICU (Fig. 1). Each new patient admission was taken as an individual entity as a source of data, meaning vital sign observation sets taken from one patient on subsequent admissions were eligible for inclusion in the analysis.

### Outcomes

We used a binary composite ‘adverse event’ outcome, which comprised in-hospital death or unplanned intensive care unit (ICU) admissions. Where patients were admitted to ICU and subsequently died, the ICU admission was taken as the event.

### Predictors

For each vital sign observation set we collected heart rate (HR), respiratory rate (RR), systolic blood pressure (SBP), peripheral blood oxygen saturation (SpO_2_), body temperature (Temp), neurological status using the Alert-Verbal-Painful-Unconscious (AVPU) scale, and the composition (air/oxygen) and delivery method (mask type) of inhaled gas. Where consciousness level had been recorded using the Glasgow Coma Scale system we converted to AVPU using a scoring system shown in the supplementary digital content **(SDC-2)**. The databases also contained survival status at hospital discharge and details of unplanned ICU admissions via linkage to electronic ICU records. We did not analyse vital sign observation sets in patients who were post-ICU admission on the general wards. In both databases the first adverse event identified was the one used in the analysis. Any complete vital sign observation sets in the 24 h preceding an event were classified as associated with an event.

### FiO_2_ calculation

FiO_2_ was taken as the prescribed value for fixed performance masks. For all other oxygen delivery systems, FiO_2_ was calculated using a published formula: FiO_2_ = (O_2_ Flow Rate + 0.21(Minute volume–O_2_ Flow Rate))/Minute volume^15^ We assumed a fixed tidal volume of 0.45 L per breath for all patients and multiplied this by the respiratory rate to obtain minute volume. A summary of the mask types and corresponding flow rates used in our calculations are shown in the supplementary digital content **(SDC-3)**. We calculated error rates associated with the assumption of a fixed tidal volume (supplementary digital content, **SDC-4**). We assumed a maximum FiO_2_ of 1.0 for all patients requiring high flow nasal oxygen and non-invasive ventilation methods.

### FiO_2_ threshold development using decision tree analysis

A Decision Tree (DT) is a predictive model that can be applied to any numeric or categorical database to establish which variables are most strongly associated with pre-specified outcomes. We adopted the methodology of Badriyah et al. to generate thresholds for the calculated FiO_2_.^16^ We used the Scikit-learn package within Python 2.7 to carry out our analysis. We generated the FiO_2_ thresholds using the OUHNHSFT database^17^ In keeping with NEWS, we assigned weights of zero, one, two and three for FiO_2_ as the concentration increased^3^, ^11^ The FiO_2_ thresholds, as well as further detail on the DT analysis, are shown in the supplementary digital content **(SDC-5, 6 and 7)**.

### Missing data

To be included in our two databases, vital sign observation sets needed to be complete with a measurement of each vital sign and the inhaled gas composition/delivery method. Fig. 1 shows the number of excluded vital sign observation sets from the analysis.

### Development databases

The OUHNHSFT database was used to derive the FiO_2_ threshold scoring bands. The QAH database was used to externally validate the NEWS-FiO_2_ score.

### Evaluating NEWS and NEWS-FiO_2_

Evaluation of NEWS and NEWS-FiO_2_ was undertaken in two stages. Firstly, we undertook the analysis on observation sets where oxygen was recorded as having been used during the admission. Secondly, we analysed score performance in all observation sets regardless of oxygen use. The primary performance measure was Area Under the Receiver Operating Characteristic curve (AUROC), which provided an overall measure of model discrimination^7^ AUROC results were reported with 95% confidence intervals, computed via bootstrapping the QAH data (through random sampling while preserving the event class prevalence of approximately 1% and repeating the test 1000 times). We used AUROC to test the ability of NEWS and NEWS-FiO_2_ to predict an adverse event up to 24 h prior. We tested the variation in AUROC for each EWS as the time-to-event window reduced from 24 h to zero. This evaluation metric showed the change in AUROC performance as the patient neared an adverse event and allowed a comparison of performance across this time frame. We used positive predictive value (PPV) vs sensitivity (also known as precision-recall) curves to show the performance of the scores rather than receiver operating characteristic curves since they de-emphasise the much greater numbers of patients without an adverse event correctly identified as true negatives. We used efficiency curves to show the number of triggers generated at different values for each score as an indication of potential workload implications on the ward. Overall sensitivity, specificity and positive predictive values were also calculated using the suggested thresholds of five or above and seven or above. It was not possible to assess calibration since NEWS does not provide estimates of absolute risk.

## Results

A flowchart of study participants is included in the supplementary digital content Fig. 1.

In the OUHNHSFT Training database there were 71,735 eligible admissions. Of those excluded, 28,750 were discharged alive in <24 h, 202 had only incomplete vital sign sets and 19 had events but no observation sets taken <24 h prior. A total of 42,764 admissions (29,931 patients) were included for analysis (the difference accounted for by multiple admissions for some patients). Of these, 17,012 admissions required oxygen therapy (14,028 patients), generating 222,156 vital sign observation sets. A total of 6469 vital sign observation sets occurred within the 24 h prior to an adverse event.

In the QAH Test database there were 250,815 eligible admissions. Of those excluded, 31,920 were discharged alive <24 h and 1532 had incomplete vital sign observation sets (there is a lower proportion of patients staying <24 h in the QAH database because of an ambulatory, short stay care facility within the OUHNHSFT) and zero had events but no observation sets taken <24 h prior. A total of 217,363 admissions (120,017 patients) were included for analysis. Of these, 83,309 admissions (57,467 patients) required oxygen, generating 1,055,423 vital sign observation sets. A total of 30,356 vital sign observation sets were tagged as associated with an adverse event.

Demographics and vital sign observation set characteristics, in both the total and oxygen requiring cohorts, are summarised in Table 1.

**Table 1:** Demographics and outcomes.

Total cohort	OUHNHSFT Training database	QAH Test database
Admissions (total)	42,764	217,363
Admissions (with >0 complete vital sign observation set)	29,931 (69.9%)	120,017 (55.2%)
Admissions with an event outcome (%)	1669 (3.9%)	7523 (3.5%)
Admissions (male) (%)	14,887 (49.7%)	56,140 (46.7%)
Admission age, mean (SD)	64(19)	63(20)
Vital sign observation sets	1201714	5545039
Vital sign observation sets tagged as event outcome (%)	9412(0.8%)	42,653 (0.8%)
Vital sign observation sets tagged as unplanned ICU outcome (%)	5503 (0.5%)	15,029 (0.3%)
Vital sign observations sets tagged as death outcome (%)	3909 (0.3%)	27,621 (0.5%)
Length of stay, median (IQR)(hours)	100 (170)	83 (143)
Heart rate, mean (SD) (beats per minute)	82 (16)	80 (16)
Respiratory rate, mean (breaths per minute)	17 (3)	17 (3)
Systolic blood pressure, mean (SD) (mmHg)	127 (22)	126 (22)
FiO_2,_ mean (SD) (%)	26 (15)	26 (12)
Body temperature, mean (degrees Centigrade)	36.4 (0.6)	36.7 (0.5)
Oxygen cohort		
Admissions (% of total cohort)	17,012 (39.7%)	83,304 (38.3%)
Admissions with an event outcome (% of oxygen cohort)	1027 (6.0%)	5688 (6.8%)
Admissions (male) (% of oxygen cohort)	8166 (48%)	37,487 (45%)
Admission age, mean (SD) (years)	65 18	68 17
Vital sign observation sets	222,156 (18.4%)	1,055,189 (19%)
Vital sign observation sets tagged as event outcome (%)	6469 (2.9%)	30,359 (2.8%)
Vital sign observation sets tagged as unplanned ICU outcome (%)	2443(1.1%)	9843 (0.9%)
Vital sign observations sets tagged as death outcome (%)	4026 (1.8%)	20,516 (1.9%)
Length of stay, median (IQR)(hours)	131 (220)	131 (213)
Heart rate, mean (SD) (beats per minute)	85 (18)	84 (18)
Respiratory rate, mean (SD)(breaths per minute)	18 (4)	18 (4)
Systolic blood pressure, mean (SD) (mmHg)	125 (23)	125 (23)
FiO_2_, mean (SD) (%)	49 (18)	47 (15)
Body temperature, mean (SD) (degrees Centigrade)	36.4 (0.7)	36.7 (0.6)

Demographic descriptors for the admissions included in each of the OUHNHSFT Training and QAH Test databases.

Table 2 shows the distributions of calculated FiO_2_ for all vital sign observation sets in the QAH Test database for the oxygen requiring cohort and categorises them into scoring thresholds. 3883 vital sign observation sets had a calculated FiO_2_ value between 21 and 22%. This occurred in patients on very low oxygen flow rates, in conjunction with higher respiratory rates (thus diluting the administered oxygen). The decision tree analysis evaluated the patients linked to these vital sign observation sets as having an equivalent risk as those not receiving oxygen, thus they scored zero points. 234,504 vital sign observation sets scored one point, 564,712 scored two points (equivalent to the score attributed in NEWS for a patient on any amount of oxygen) and 252,090 scored three points. Overall, 46.5% of the vital sign observation sets scored zero, one or three points and 53.5% scored two points.

**Table 2:** FiO_2_ scoring bands statistics in the QAH Test database for patients receiving oxygen therapy.

Score	0	1	2	3	Sum
FiO_2_ thresholds (%)	21–22	22.1–37	37.1–53	>53	
Vital sign observation sets	3883	234,504	564,712	252,090	1,055,189
Vital sign observation sets tagged as an event	56	6779	9066	14458	30,359

Analysis on the FiO2 scoring bands statistics. The table summarises how oxygenated patient observations are clustered in each of the proposed FiO2 bands.

**Fig. 2:**
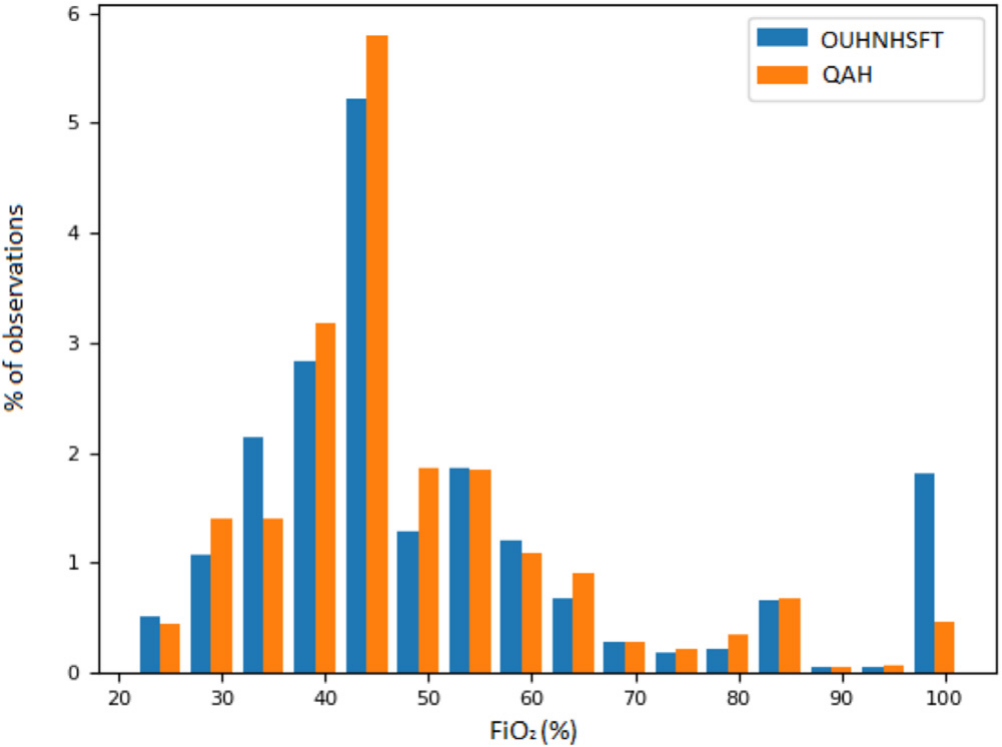
FiO_2_ histogram of the oxygen requiring cohort in both databases. QAH: Queen Alexander Hospital, OUHNHSFT: Oxford University Hospitals National Health Service Foundation Trust. Percentage total observations (both oxygen requiring and non-oxygen requiring vital sign observation sets), divided into 5%. from 25 to 100%.

Fig. 2 shows the distribution of FiO_2_ concentrations in both the OUHNHSFT and QAH. We report all inspired oxygen concentrations as percentages. We report the distribution of calculated FiO_2_ values (for each database) as a percentage of the total cohort of vital sign observation sets. FiO_2_ concentrations less than 25% were not shown in the figure because the group’s disproportionate height made it difficult to represent graphically with the other groups. The most common FiO_2_ in both databases was 45%, each accounting for >5% of the total vital sign observation sets. The higher percentage of vital sign observation sets with a FiO_2_ of 100% in the OUHNHSFT is accounted for by the higher provision of non-invasive and nasal-high-flow cannula ventilation strategies on the wards of this Trust.

### Performance of the early warning scores

Table 3 shows the observation level AUROC (with 95% CI) for each scoring system against an outcome of ‘adverse event’ in the subsequent 24 h. It also shows the sensitivity, specificity, and positive predictive value of the scores for thresholds of ≥5 and ≥7 respectively. In the oxygen requiring cohort, NEWS-FiO_2_ (AUROC of 0.823, 95% CI 0.819−0.824) out performed NEWS (AUROC of 0.811, 95% CI 0.809−0.814) when predicting in-hospital death or unplanned ICU admission within 24 h of the observation set. NEWS-FiO_2_ also outperformed NEWS in sensitivity, specificity and positive predictive value when using five and seven as trigger thresholds. In terms of admission level performance, NEWS-FiO_2_ identified an additional 173 admissions (out of the total 83,304 in the oxygen therapy cohort) who went on the have an adverse event.

**Table 3:** Performance of NEWS and NEWS-FiO_2_ in *oxygen requiring* cohort in the QAH Test database.

	NEWS	NEWS-FiO_2_
AUROC (CI)	0.811 (0.809–0.814)	0.823 (0.819–0.824)
Sensitivity (%) (Score ≥5/≥7)	81.4/56.9	82.7/60.2
Specificity (%) (Score ≥5/≥7)	64.7/87.5	64.8/87.6
Positive Predictive Value (%) (Score ≥5/≥7)	6.4/11.9	6.5/12.5
Efficiency (%) (Score ≥5/≥7)	36.6/13.7	36.5/13.7

Performance metrics of the scoring systems (NEWS, NEWS-FiO2) for predicting the event outcome in the QAH Test database, which includes the Area Under the Receiver Operating Characteristic curve (AUROC), with 95% confidence interval (CI), and sensitivity, specificity and positive predictive value values at a threshold of 5 and 7.

**Fig. 3:**
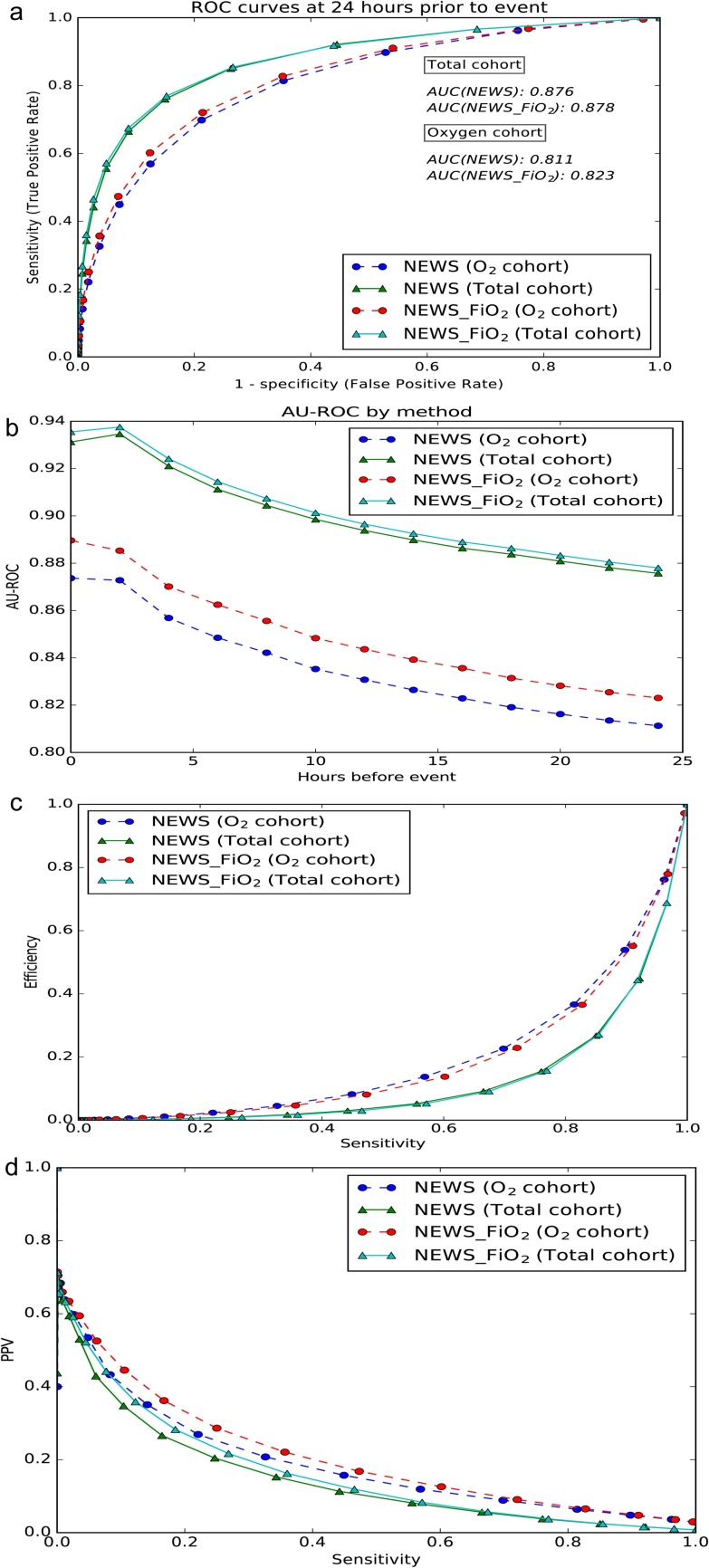
(a) Receiver Operating Characteristic curve (ROC) for NEWS and NEWS-FiO_2_ om oxygen requiring and total patient cohorts. (b) Area Under the Receiver Operating Characteristic (AUROC) performance when time-to-event approaches the event time for NEWS and NEWS-FiO_2_ in the oxygen requiring and total patient cohorts. (c) Efficiency curves for NEWS and NEWS-FiO_2_ in oxygen requiring and total patient cohorts. The curve shows the fraction of the total number of observations at, or above, each EWS value against the fraction of the total observations for which the event outcome was true at, or above, that EWS value (d) Precision-recall curves for NEWS and NEWS-FiO_2_ in the oxygen requiring and total patient cohorts.

Fig. 3(a) displays the AUROC curves for each score in both the oxygen requiring cohort and the total cohort. Fig. 3(b) shows improving performance in both EWS in AUROC over time as observation sets approach the adverse event. The FiO_2_ enhanced score outperforms the non-enhanced score throughout and particularly in the oxygen requiring cohort. Efficiency curves are displayed in Fig. 3(c) but do not show any obvious performance gains, potentially as a result of the small fraction of true positive results within the entire database. Fig. 3(d) shows the precision-recall curves.

## Discussion

### Statement of key findings

In ward patients requiring oxygen therapy, NEWS-FiO_2_ outperformed NEWS when predicting in-hospital death or unplanned ICU admission within 24 h. Our results support the hypothesis that introducing FiO_2_ thresholds to increase the granularity of oxygen therapy scores from a binary system (on/off oxygen), improved the sensitivity and positive predictive value for a similar number of escalations (workload). These findings translate into the following observation level findings (using a threshold of ≥7): The workload was the same (137 alerts per 1000 vital sign observation sets). NEWS-FiO_2_ increased the positive predictive value (an additional six adverse event per 1000 alerts) and the sensitivity (an additional 33 alerts per 1000 adverse events). At the admission level (using a threshold of ≥7): there were a total of 83,304 admissions in the oxygen therapy cohort. In this cohort NEWS-FiO_2_ would have correctly identified an additional 173 individual admissions who went on to have an adverse event.

### Comparison to previous studies

To our knowledge, no widely used general adult EWS includes FiO_2_ as a predictor variable.^18^, ^19^ Carle et al. designed and internally validated an obstetric EWS using the FiO_2_ required to maintain SpO_2_ > 96% as a variable.^9^ However this EWS has not been translated into widespread use. We adopted the machine learning, decision tree methodology of Badriyah et al., who produced the first decision tree EWS (DTEWS).^20^ Ours is the first study to use decision tree analysis for the derivation of thresholds for FiO_2._ It is also the first study to use a machine learning method to add a variable to NEWS and evaluate its effect on performance. The relatively modest performance gain achieved by adding FiO_2_ is comparable to previous studies that evaluated adding individual vital signs to EWS systems.^21^

### Implications for clinicians and policy makers

EWS systems are well established in the UK, with the heuristically developed NEWS being used in 75% of NHS hospitals.^10^, ^12^ Since then, digital EWS platforms have been developed, meaning complex algorithms using vital sign observation sets can be introduced without increasing calculation error.^14^, ^22^ NEWS2 is a new score being adopted nationally in the UK. It is specifically designed to improve EWS performance in patients with hypercapnic respiratory dysfunction.^10^ NEWS2 emphasises the interrelationship between oxygen therapy (or lack thereof) and harm in high risk patient groups. We propose quantifying oxygen therapy via FiO_2_ and evaluating the associated relationships with adverse events may be a logical first step in evaluating this important research question.

### Limitations

Assumptions in the FiO_2_ derivation formula led to some minor but systemic error in the calculation of FiO_2_ across the patient cohort. This error is clarified in detail in the supplementary digital content **(SDC-7)**. We also acknowledge that not all patients on high-flow nasal prong oxygen therapy or Non-Invasive Ventilation modes will achieve a FiO_2_ of 1.0. However, this will not have affected the score performance because the lower limit for the high scoring FiO_2_ band was 53%. This assumption introduced some error to the analysis. Using death or unplanned ICU admission within 24 h as an outcome measure was in accordance with similar research. However, this outcome measure has limitations. We did not have the data to exclude patients on ‘end of life’ pathways. Confounding will occur in retrospective, observational data analysis in patient populations where EWS systems are in use. In this study, scoring for oxygen therapy using NEWS increased the risk score, potentially above the alerting threshold. This should have activated a clinical review, potentially facilitating the patient avoiding an adverse event. This trigger in turn becomes a false positive result and reduces the AUROC^23^, ^3^ Finally, by deriving and testing EWS systems in databases derived from hospitals with EWS in place, the study was seeking to demonstrate incremental gains, which may have been more difficult to detect. A combination of all these factors could explain the modest performance gain seen from NEWS-FiO_2_ to NEWS and merit further investigation.

### Strengths

Our study is the first to use an automated process such as decision tree analysis to introduce an additional variable to an EWS in a data driven way. We evaluated the effect in a totally separate patient population. We show that a more a granular score for oxygen therapy improves EWS performance. By using the TRIPOD guidelines, we adhered to best practice and ensured the reporting of methods and results are transparent and robust^24^ The recent introduction of NEWS2 makes the timing of this study important.^10^

### Future work

Further research is needed to evaluate the relationship between FiO_2_ and SpO_2_ and their combined associations with adverse events in ward patients, in patients with and without chronic pulmonary disease.

## Conclusion

Our study demonstrates that decision tree analysis is an effective method when adding FiO_2_ to NEWS in a data driven way. In the ≈40% of ward patients requiring oxygen therapy, NEWS-FiO_2_ outperformed NEWS when predicting in-hospital death or unplanned ICU admission in the next 24 h. Adding FiO_2_ to NEWS (and other EWS) warrants further study, particularly in patients with or at risk of respiratory dysfunction.

## Data sharing

The raw data used in this research are not openly available.

## Trial registration

Health Research Authority approval was obtained for gathering the data used in this study from the Research Ethics Committees (REC reference: Oxford 16/SC/0264, Isle of Wight, Portsmouth and South East Hampshire 08/02/1394) and confidentiality advisory group (16/CAG/0066).

## Disclaimers

The views expressed in this study are those of the authors and not the official position of the University of Oxford, the Department of Health & Social Care or the Wellcome Trust.

## Financial support

This publication presents independent research funded by the Health Innovation Challenge Fund (HICF-R9-524; WT-103703/Z/14/Z), a parallel funding partnership between the Department of Health and Wellcome Trust. The views expressed in this publication are those of the authors and not necessarily those of the Department of Health & Social Care or Wellcome Trust. PW is supported by the National Institute for Health Research Biomedical Research Centre, Oxford.

## Conflicts of interest

None.

## Study design

PJW, NF, JM; data collection: MP, PM; data analysis: NF, JM, OR; data interpretation and writing the paper: all authors contributed.
