# Supplementary material for: The effect of fractional inspired oxygen concentration on early warning score performance: A database analysis
Source: Resuscitation. 2019 Jun;139:192–9. doi: 10.1016/j.resuscitation.2019.04.002 (PMC6547016; doi:10.1016/j.resuscitation.2019.04.002)
Supplement: Supplementary file 1 [file mmc1.pdf]

SDC-1 TRIPOD checklist

Section/Topic	Item		Checklist Item	Page
Title and abstract				
Title	1	D;V	Identify the study as developing and/or validating a multivariable prediction model, the target population, and the outcome to be predicted.	title
Abstract	2	D;V	Provide a summary of objectives, study design, setting, participants, sample size, predictors, outcome, statistical analysis, results, and conclusions.	abstract
Introduction				
Background and objectives	3a	D;V	Explain the medical context (including whether diagnostic or prognostic) and rationale for developing or validating the multivariable prediction model, including references to existing models.	Intro. P2
	3b	D;V	Specify the objectives, including whether the study describes the development or validation of the model or both.	Intro. P3
Methods				
Source of data	4a	D;V	Describe the study design or source of data (e.g., randomized trial, cohort, or registry data), separately for the development and validation data sets, if applicable.	Meth.P1
	4b	D;V	Specify the key study dates, including start of accrual; end of accrual; and, if applicable, end of follow-up.	SrcData.P1
Participants	5a	D;V	Specify key elements of the study setting (e.g., primary care, secondary care, general population) including number and location of centers.	Part. P1
	5b	D;V	Describe eligibility criteria for participants.	Part. P2
	5c	D;V	Give details of treatments received, if relevant.	NA
Outcome	6a	D;V	Clearly define the outcome that is predicted by the prediction model, including how and when assessed.	Outcomes
	6b	D;V	Report any actions to blind assessment of the outcome to be predicted.	NA
Predictors	7a	D;V	Clearly define all predictors used in developing or validating the multivariable prediction model, including how and when they were measured.	Pred
	7b	D;V	Report any actions to blind assessment of predictors for the outcome and other predictors.	FiO2. P2
Sample size	8	D;V	Explain how the study size was arrived at.	SDC-3
Missing data	9	D;V	Describe how missing data were handled (e.g., complete-case analysis, single imputation, multiple imputation) with details of any imputation method.	MissingData
	10a	D	Describe how predictors were handled in the analyses.	DT

Statistical analysis methods	10b	D	Specify type of model, all model-building procedures (including any predictor selection), and method for internal validation.	DT
	10c	V	For validation, describe how the predictions were calculated.	DT & FiO2
	10d	D;V	Specify all measures used to assess model performance and, if relevant, to compare multiple models.	Eval.P 1
	10e	V	Describe any model updating (e.g., recalibration) arising from the validation, if done.	NA
Risk groups	11	D;V	Provide details on how risk groups were created, if done.	Eval.P 2
Development vs. validation	12	V	For validation, identify any differences from the development data in setting, eligibility criteria, outcome, and predictors.	NA
Results				
Participants	13a	D;V	Describe the flow of participants through the study, including the number of participants with and without the outcome and, if applicable, a summary of the follow-up time. A diagram may be helpful.	Res.P 1
	13b	D;V	Describe the characteristics of the participants (basic demographics, clinical features, available predictors), including the number of participants with missing data for predictors and outcome.	Res.P 1
	13c	V	For validation, show a comparison with the development data of the distribution of important variables (demographics, predictors and outcome).	Res.P 2
Model development	14a	D	Specify the number of participants and outcome events in each analysis.	Res.P 1,2
	14b	D	If done, report the unadjusted association between each candidate predictor and outcome.	NA
Model specification	15a	D	Present the full prediction model to allow predictions for individuals (i.e., all regression coefficients, and model intercept or baseline survival at a given time point).	Res.P 3
	15b	D	Explain how to the use the prediction model.	Res.P 3
Model performance	16	D;V	Report performance measures (with CIs) for the prediction model.	Perf.P 1
Model-updating	17	V	If done, report the results from any model updating (i.e., model specification, model performance).	NA
Discussion				
Limitations	18	D;V	Discuss any limitations of the study (such as nonrepresentative sample, few events per predictor, missing data).	*Limit*
Interpretation	19a	V	For validation, discuss the results with reference to performance in the development data, and any other validation data.	Dis.P 1,2,3

				+ Comp
	19b	D;V	Give an overall interpretation of the results, considering objectives, limitations, results from similar studies, and other relevant evidence.	Dis.P 1,2,3 + Stren
Implications	20	D;V	Discuss the potential clinical use of the model and implications for future research.	DisP,I mpl
Other information				
Supplementary information	21	D;V	Provide information about the availability of supplementary resources, such as study protocol, Web calculator, and data sets.	*Supp l*
Funding	22	D;V	Give the source of funding and the role of the funders for the present study.	*Fund*

SDC-2 GCS (Glasgow Coma Scale) converted to AVPU (Alert Verbal Pain Unresponsive) scale following the table below.

GCS	AVPU
>=15	1 (Alert)
12 - 15	2 (Verbal)
8-12	3 (Pain)
<=8	4 (Unresponsive)

GCS to AVPU conversion parameters.

SDC-3 Mask types and corresponding flow rates

For the documentation of the oxygen delivery system, the device codes within both NHS trusts are aligned according to BTS guideline recommendations.

BTS codes	OUHT database		Mask Prevalence (100)	PHT database		Mask Prevalence (%)
	mask name	coded value (%)		mask name	Coded value (%)	
A	Room Air	21		Room Air	21	
V	Venturi oxygen 24%	24	1.6	Venturi (variable)	R/Rand***	11.2

V	Venturi oxygen 28%	28	1.8			
V	Venturi oxygen 35%	35	1.3			
V	Venturi oxygen 40%	40	0.6			
V	Venturi oxygen 60%	60	0.3			
H	Humidified oxygen 28%	28	0.7			
H	Humidified oxygen 35%	35	0.5			
H	Humidified oxygen 40%	40	0.3			
H	Humidified oxygen 60%	60	0.4	Humidified (60% max)	60	4.3
H	Humidified oxygen 80%	80	0.1			
H	Humidified oxygen 98%	98	0.1	Humidified (98% max)	98	0.0
N	Nasal cannula	converted*	71.4	Nasal cannula	converted*	72.6
SM	Simple mask	converted*	11.6	Simple Mask	converted*	7.0
RM	Reservoir mask	80	2.2	Reservoir	80	2.7
TM	Tracheostomy mask	converted*	0.3	Tracheostomy / Trach flow	R/converted*	0.4
CP	CPAP+	100	0.6	CPAP+ / BIPAP++	100	1.1
NIV	Non-invasive	100	1.6	NIV+++	100	0.4
OTH	Other device	converted*	0.0	Aerosol	converted*	0.3
SM	Nebuliser Mask	converted*	0.03	Nasal humidified	converted*	0.0
HFN	High Flow / Optiflow	100	4.7	Intubated Conc./Flow	100	0.002

Mask types and corresponding flow rates in percentage, from each trust: Oxford University Hospitals NHS Foundation Trust (OUHNHSFT) and Portsmouth Hospitals Trust (PHT).

** converted refers to the use of the Bateman equation for computing the FiO_2 value.*

*** R/converted refers to conditions where the O_2 flow rate has been recorded in a mixed and potentially inaccurate manner. In these circumstances, we made the assumption that O2 flows recorded > 21 were in fact FiO2 recordings and we analysed those values as percentage FiO2 values. For any values recorded as < 21, we used the Bateman equation for converting the O_2 Flow rate to FiO_2.*

*R/Rand*** refers to conditions where the O_2 flow rate has been recorded in a mixed and potentially inaccurate manner. In these circumstances, we first checked whether the value reported as O_2 flow rate was > 21. If so, the value was considered as the mistakenly reported FiO_2 and analysed as such. For any values < 21, we randomly generated a percentage in the valid range for Venturi masks (24%-60%).*

+ *CPAP: Bilevel Positive Airway Pressure mask*

++ *BIPAP: Continuous Positive Airway Pressure mask*

+++ *NIV: Non-Invasive Ventilation mask*

SDC-4: Error rates for different flow rates and tidal volumes

Ratio of O_2 Flow rate to Respiratory Rate	Tidal Volume (liter)	FiO_2 (%)
	0.4	1.00
0.5	0.45	1.00
	0.6	0.87
	0.4	1.00
0.4	0.45	0.91
	0.6	0.74
	0.4	0.80
0.3	0.45	0.74
	0.6	0.60
	0.4	0.60
0.2	0.45	0.56
	0.6	0.47
	0.4	0.41
0.1	0.45	0.39
	0.6	0.34
	0.4	0.23

0.01	0.45	0.23
	0.6	0.22

Error rates in calculating FiO_2. O_2 flow rate/respiratory rate ratio values are combined with different tidal volume assumptions (0.45, 0.4 and 0.6) to produce different $Fi0_2$ values [$FiO_2 = (0.21 + 0.79*R/TV)$]. We used a fixed tidal volume of 0.45 (liter per breath) for all patients. The results show the variation in FiO_2 for patients weighing between 57kg (Vt of 0.4) and 85kg (Vt of 0.6) based on a tidal volume of 7mls/kg. Pragmatically, bigger, faster breaths reduce FiO_2 because they dilutes any supplemental oxygen being administered.

SDC-5 Comparison of scoring systems

Vitals	EWS	3	2	1	0	1	2	3
RR	NEWS	< 8		9 - 11	12 - 20		21 - 24	> 24
HR	NEWS	<40		41-50	51-90	91-110	111-130	>131
SBP	NEWS	<90	91 -100	101-110	111-219			>219
SpO_2	NEWS	<91	92-93	94-95	>96			
Temp	NEWS	< 35		35.1 - 36	36.1 - 38	38.1 - 39	>39.1	
FiO_2	NEWS		Yes		No			
	NEWS-FiO_2				0-22	22.1-37	37.1-53	>53
AVPU	NEWS				A			V,P,U

NEWS: National Early Warning Score Decision Tree Early Warning Score of Oxford, where the scoring bands are derived using the OUHNHSFT Training dataset collected at the Oxford University Hospitals Trust.

SDC-6 Decision Tree Algorithm

The decision tree is a graphical model that is composed of a set of nodes and edges which are organised in hierarchical structure. The tree originates from a root node which contains the whole data. The distribution of the labelled samples (observations recorded within the 24 hours preceding an event) at the root node are divided into two subsets, using a decision function. The aim is to search for the best feature and the best split in that feature's range which maximizes the data purity in the subsequent subsets. Denoting the training data as $(\mathbf{x}_i; y_i) = (x_1, x_2, ..., x_d, y_i)$, a decision tree recursively partitions the space such that the samples with the same labels are grouped together. Representing the data at node n by Q. For each candidate $split = (j, t_m)$ consisting of a feature j and threshold t_m, the algorithm partitions the data into $Q_{left}(\vartheta)$: $(\mathbf{x}; y)|x_j <= t_m$ and $Q_{right}(\vartheta) : Q \backslash Q_{left}(\vartheta)$ subsets. The impurity at node n can then be formulated as:

$$G(Q, \vartheta) = (n_{left}/N_n)\ H(Q_{left}(\vartheta)) + (n_{right}/N_n)\ H(Q_{right}(\vartheta))$$

where $H(Xn) = \Sigma k\ p_n k\ (1 - p_n k)$ is known as the Gini impurity measure and k denotes the number of classes. The aim of the partitioning is then to select the parameters that minimises the impurity:

$\vartheta^* = argmin\ \vartheta\ G(Q, \vartheta)$

The algorithm recursively partitions data in hierarchical fashion for subsets $Q_{left}(\vartheta^*)$ and the algorithm recursively partitions data in hierarchical fashion for subsets $Q_{left}(\vartheta^*)$ and $Q_{right}(\vartheta^*)$ until the maximum allowable depth or the minimum number of node samples is reached (this is set to ten samples in our implementation). This in practice in a uni-variate tree, infers that the same feature will be recursively partitioned at different thresholds until one of the stopping criteria is reached. Once a decision tree is formed for a vital sign data i.e. RR, each node in the tree is assigned with a risk value that is estimated as the proportion of the number of observations followed by an abnormal event to the total number of observations reaching that node. The conversion of the node risk values to weighting scores is then undertaken following Algorithm1: where the nodes with a node risk value less than mean risk (μr), was ascribed the value 0 , the node risk values greater than the mean risk and less than two times the mean risk were ascribed value 1, the node risk values greater than two times the mean risk and less than three times the mean risk are ascribed value 2 and finally if the node risk value is greater than three times the mean risk, it was ascribed to value 3.

Algorithm 1: DTEWS weight association algorithms

Require: $\mu_r \to |N^{event}|\backslash|N^{Total}|$
Require: treeRisks -> {nodeRisk(n)}, n N ode where N ode is set of tree nodes and nodeRisk(n) = $|N_n^{event}|\backslash|N_n^{Total}|$ with
N_n representing the number of samples at node n in the tree.
for i, nodeRisk in enumerate(treeRisks):
if (nodeRisk < μ_r):
nodeScore[i] = 0
else if (μ_r < nodeRisk < 2 μ_r):
nodeScore[i] = 1
else if (2 μ_r <nodeRisk < 3 μ_r):
nodeScore[i] = 2
else if (nodeRisk> 3 μ_r):
nodeScore[i] = 3
end

SDC-7: FiO_2 scoring algorithm

Step 1. Align the trust-dependent Oxygen mask coding system with the BTS standard

Step 2. Use the coding system introduced in SDC-5 to compute a mask-independent FiO_2 value in percentage

Step 3. Use the FiO_2 thresholding values (shaded in green) introduced the SDC-3 Table to compute an EWS score for each recorded value

Step 4. Aggregate the score computed for the FiO_2 observations in Step 3 with scores computed for all other vital observations in each EWS-FiO_2 model
